# Therapeutic potential of *Litsea cubeba* essential oil in modulating inflammation and the gut microbiome

**DOI:** 10.3389/fmicb.2023.1233934

**Published:** 2023-08-14

**Authors:** Liqiong Xia, Ran Li, Ting Tao, Ruimin Zhong, Haifang Du, Ziling Liao, Zhanghua Sun, Changqiong Xu

**Affiliations:** ^1^Department of Pharmacy, Loudi Central Hospital, Loudi, Hunan, China; ^2^Guangdong Provincial Key Laboratory of Utilization and Conservation of Food and Medicinal Resources in Northern Region, Shaoguan University, Shaoguan, Guangdong, China; ^3^Hunan Yueyang Maternal and Child Health-Care Hospital, Yueyang, Hunan, China; ^4^The Second Clinical Medical College, Guangzhou University of Chinese Medicine, Guangzhou, China; ^5^Medical College of Shaoguan University, Shaoguan, Guangdong, China

**Keywords:** inflammation, essential oils, gut microbiome, LPS, *Litsea cubeba*, citral

## Abstract

Inflammation, a sophisticated and delicately balanced physiological mechanism, is paramount to the host’s immunological defense against pathogens. However, unfettered and excessive inflammation can be instrumental in engendering a plethora of chronic ailments and detrimental health repercussions, notably within the gastrointestinal tract. Lipopolysaccharides (LPS) from bacteria are potent endotoxins capable of instigating intestinal inflammation through the disruption of the intestinal epithelial barrier and the stimulation of a pro-inflammatory immune response. In this study, we sought to investigate the influence of *Litsea cubeba* essential oil (LCEO) on LPS-induced intestinal inflammation and associated changes in the gut microbiota. We investigated the therapeutic potential of LCEO for gut health, with particular emphasis on its gut protective properties, anti-inflammatory properties and modulation of the gut microbiome. LCEO exhibited protective effects on colonic tissue by protecting crypts and maintaining epithelial integrity, and anti-inflammatory properties by reducing TNF-α, IL-6, and IL-1β levels in the liver and intestine. Citral, a major component of LCEO, showed robust binding to IL-1β, IL-6, and TNF-α, exerting anti-inflammatory effects through hydrogen bonding interactions. Using community barplot and LEfSe analyses, we detected significant variation in microbial composition, identified discrete biomarkers, and highlighted the influence of essential oils on gut microbial communities. Our research suggests that LCEO may be a promising natural compound for ameliorating diarrhea and intestinal inflammation, with potential implications for modulating the gut microbiome. These observations provide invaluable insight into the potential therapeutic role of LCEO as a natural anti-inflammatory agent for treating intestinal inflammatory disorders, particularly in the setting of a dysregulated immune response and altered gut microbiota. Furthermore, our findings highlight the need to understand the complex interplay between the host, the gut microbiome and natural products in the context of inflammatory diseases.

## 1. Introduction

Inflammation manifests as a physiological response to injury or infection, serving as a pervasive pathological mechanism underlying a myriad of diseases. The immune system, primarily mediated by macrophages, releases inflammatory mediators, including nitric oxide (NO), tumor necrosis factor-α (TNF-α), interleukin-6 (IL-6), and interleukin-1β (IL-1β), in its endeavor to combat pathogens. However, an exaggerated inflammatory response can give rise to acute and chronic conditions and, in severe cases, can precipitate shock or even mortality (Barton, 2008). Bacterial lipopolysaccharides (LPS), which constitute the outer membrane of Gram-negative bacteria, exert profound pro-inflammatory effects and have been implicated in gut inflammation (Candelli et al., 2021). LPS have demonstrated its capacity to instigate intestinal damage across diverse animal models through the upregulation of pro-inflammatory mediators and the imposition of oxidative stress (Wu et al., 2018; Zhu et al., 2018; Zhuang et al., 2019). Some investigations have proposed that excessive immune reactions to pathogens, characterized by an amplified release of pro-inflammatory cytokines, can impede the functionality of the intestinal barrier, resulting in increased epithelial apoptosis and reduced expression of junctional proteins. Notably, the gut microbiome serves as a substantial reservoir of LPS in both humans and animals. Previous studies have elucidated that alterations in the microbial composition of the host’s gut can induce persistent intestinal inflammation and metabolic dysfunction (Sommer and Backhed, 2013). Currently, there are three primary categories of conventional medications used in the management of inflammatory bowel disease, namely aminosalicylic acid derivatives, glucocorticoids, and immunosuppressants. Nonetheless, the necessity for novel therapeutic alternatives arises from the potential long-term adverse effects associated with these treatments. Indeed, herbal medicine is extensively applied worldwide for the treatment of inflammatory bowel disease, and substantial progress has been achieved in both clinical and fundamental research in recent decades (Zheng et al., 2021).

Essential oils (EOs) are a diverse group of volatile compounds synthesized by aromatic plants as secondary metabolites. They occur in various plant organs and are primarily intended to shield plants from aggression by bacteria, fungi, and viruses, as well as insects. There are vast amounts of EOs from different plants worldwide, most of which have been at least partially characterized for their antimicrobial activity against Gram-positive and Gram-negative bacteria, fungi, and viruses. The composition of EOs was chosen by nature over millions of years of evolution, during which, competitive selection processes acted on their antibacterial, antifungal, and antiviral activities in an evolutionary conflict between plant survival and microbial aggressions. EOs have adopted multi-target mechanisms of action that make it challenging for microorganisms to develop resistance to these compounds, making them a promising therapeutic intervention (Sharma et al., 2019). EOs and their molecules are capable of modulating various signaling pathways that are dysregulated during acute or chronic inflammatory responses. The complex relationships between the human intestinal microbiota, consisting of bacteria, fungi, and viruses, and its intricate pathophysiological interactions with the immune system and the enteric nervous system make EOs particularly intriguing for their antimicrobial activity, which is often selective for different microbial components. From this viewpoint, EOs have the potential to be potent modulators of the intestinal microbiota (De Fazio et al., 2016).

Among the plants that produce EOs, *Litsea cubeba*, also known as Lour. Pers. (Lauraceae), is a species widely distributed across the eastern and southern regions of China and other parts of Southeast Asia, is renowned for its essential oil, which is extracted from the plant’s fruits and has a distinctive crisp lemon aroma. This oil has a wealth of potential health benefits, including anti-inflammatory, antimicrobial and antioxidant properties (Feng et al., 2009). In recent years, a growing number of studies have reported that extracts and compounds derived from *L. cubeba* possess a diverse range of pharmacological properties, including anticancer, antibacterial, antioxidant, and anti-inflammatory activities (Lin et al., 2013). One of the most notable bioactive components of *L. cubeba* essential oil (LCEO) is citral, which is widely used in a variety of industries, including medicine, food, and chemicals. Despite the growing interest in *L. cubeba* and its potential therapeutic properties, the effects of LCEO on intestinal inflammation remain largely unknown. Therefore, further research is required to investigate the efficacy of LCEO and its potential to modulate the intestinal microbiota.

In this study, we endeavored to explore the impact of *L. cubeba* essential oil (LCEO) on LPS-induced intestinal inflammation and the consequent alterations in the gut microbiome. Our investigation divulged that LCEO administration effectively alleviated LPS-induced gut inflammation, thereby substantiating its potential therapeutic utility in the management of intestinal inflammatory diseases. Furthermore, our findings suggest that LCEO’s anti-inflammatory action is intricately linked to its modulatory effects on the composition and diversity of the gut microbiota. These insightful results bolster the notion that LCEO could serve as a promising candidate for the treatment of intestinal inflammation, and potentially other gastrointestinal ailments that afflict the human population.

## 2. Methods

### 2.1. Chemicals

The essential oil of *Litsea cubeba* was procured from Jiangxi Baicao Pharmaceutical Co., Ltd. The lipopolysaccharides (LPS) were obtained from Beijing Solarbio Science & Technology Co. Ltd. The genes for IL-6, IL-1β, and TNF-α were acquired from Sangon Biotech Co., Ltd. The Trizol and RT Mix Kit with gDNA Clean for qPCR were supplied by Accurate Biology. The SYBR Green reagent was obtained from Roche. The C57BL/6J and SPF chow were obtained from Changsha Tianqin Biotechnology Co. Ltd. All other chemicals utilized were of the highest analytical grade.

### 2.2. Experimental design and induction of colitis

**Figure 1:**
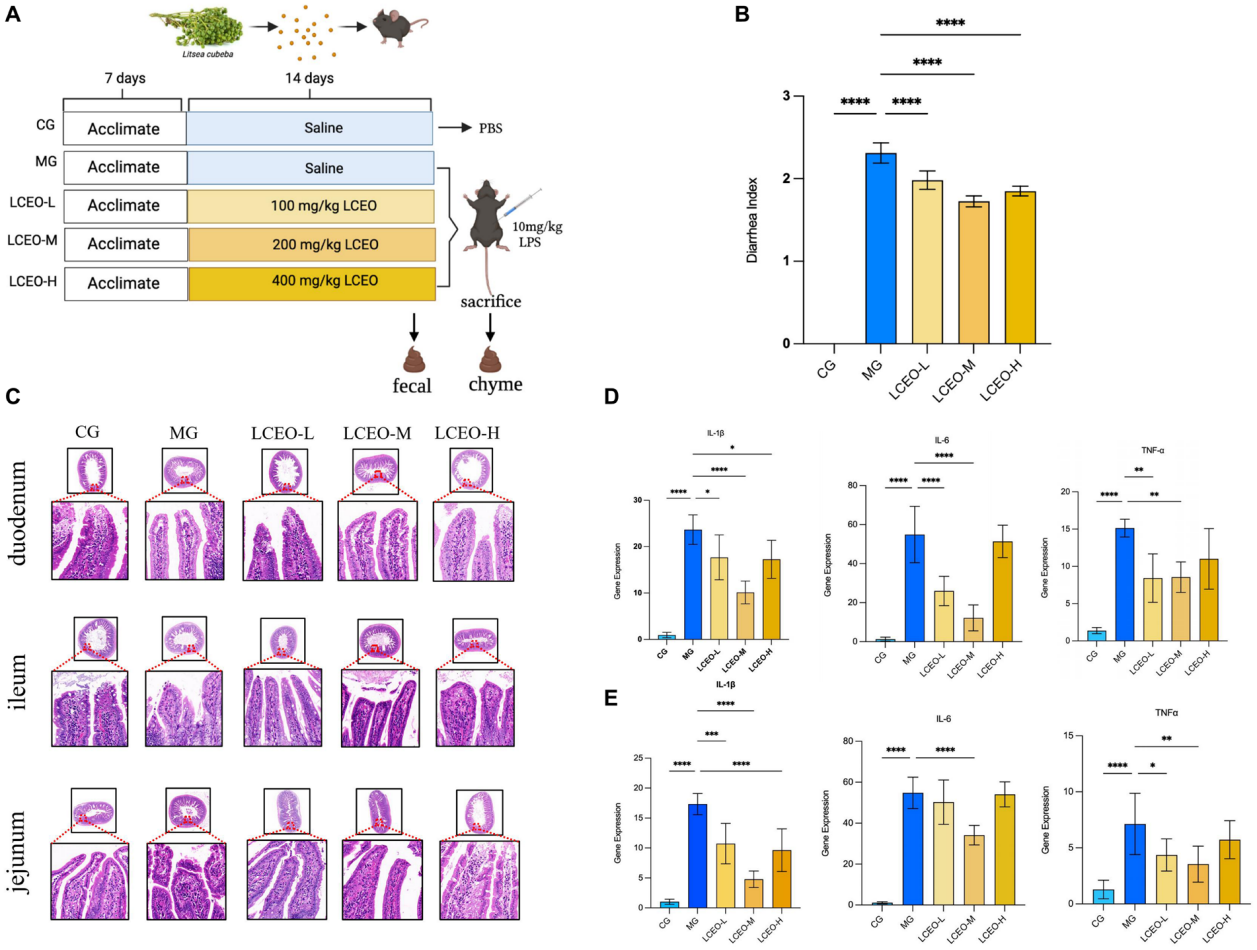
**(A)** Experimental design in mice. **(B)** The effect of LCEO of DAI index in all groups. **(C)** Pathological sections of the intestinal of various groups of mice (20×). **(D)** Effect of LCEO on the level of liver inflammatory cytokines. **(E)** Effect of LCEO on the level of duodenum inflammatory cytokines. *p < 0.05, **p < 0.01, ***p < 0.005, ****p < 0.001.

In accordance with the established guidelines of the Animal Ethics Committee at Hunan Heyuan Biotechnology Co., Ltd. (LCH2022015), a cohort of 50 male C57BL/6J mice was acquired from Hunan SJA Laboratory Animal Co. The mice, which weighed 18–20 grams and were 8 weeks old, were randomly assigned to five groups [control, model, low-dose LCEO (LCEO-L, 100 mg/kg), medium-dose LCEO (LCEO-M, 200 mg/kg), and high-dose LCEO (LCEO-H, 400 mg/kg)], and housed in a controlled environment characterized by consistent temperatures, humidity, and light–dark cycles. On the 21nd day, the fresh fecal samples were collected to assess the safety and efficacy of LCEO by microbiota analysis. On the 22nd day, the control group was administered PBS, while the other groups were given an intraperitoneal injection of LPS at a dose of 10 mg/kg. Following a 6-h fast, the mice were weighed and then sacrificed to obtain the tissues of liver, duodenum, jejunum, ileum and intestinal contents for further biochemical and microbiota analysis. All collected samples were stored at −80°C (Figure 1A).

### 2.3. Molecular docking simulation

A molecular docking simulation was performed by downloading the crystal structure of the genes from RCSB Protein Data Bank30 and the small molecules from TCMSP MENU. Autoduck software was used for molecular docking, and PyMol2.3.0 software was used for visualizing the binding model. In summary, this study used various tools and databases for prediction of the targets, protein–protein interaction analyses, KEGG pathway analyses, GO functional analyses, construction of the network, and the molecular docking simulation. The results provided valuable insights into the molecular mechanisms underlying the effects of compounds on the targets of citral related to inflammation.

### 2.4. Anti-inflammatory gene expression levels determined via RT-qPCR

RNA was extracted from the liver and duodenum using Trizol reagent. Primers were specially designed for the RT-qPCR analysis of IL-1β, TNF-α, and IL-6. The RT-qPCR assay was performed using an analytical qTOWER3G instrument (Germany), with a reaction volume of 10 μL, including 1 μL of the cDNA template and 5 μL of SYBR^®^ Green real-time PCR master mix (solarbio, China). The PCR protocol consisted of an initial denaturation phase at 95°C for 10 min, followed by 40 cycles of denaturation at 95°C for 15 s, annealing at 60°C for 60 s, and a dissociation phase comprising denaturation at 95°C for 10 s, annealing at 65°C for 1 min, extension at 97°C for 1 s, and cooling at 37°C for 1 min. The threshold cycle values for each gene were obtained, and the genes’ expression levels were calculated using the 2^−ΔΔCT^ method. The primer sequences are provided in Table 1.

**Table 1:** Sequences of primers used for real-time polymerase chain reaction.

Gene	Accession number	Primer sequence (5′–3′)
GAPDH	NM_008084.2	CCTCGTCCCGTAGACAAAATG TGAGGTCAATGAAGGGGTCGT
TNF-α	NM_013693.3	ATGTCTCAGCCTCTTCTCATTC GCTTGTCACTCGAATTTTGAGA
IL-6	NM_001314054.1	AGACTTCCATCCAGTTGCCT CAGGTCTGTTGGGAGTGGTA
IL-1β	NM_008361.4	ACTCATTGTGGCTGTGGAGA TTGTTCATCTCGGAGCCTGT

### 2.5. Histological studies

The duodenum, jejunum, and ileum from the mice were subjected to a washing procedure using phosphate-buffered saline (PBS), followed by fixation in 4% formaldehyde for a duration of 1 h at 4°C. The tissues underwent dehydration through sequential immersion in solutions of alcohol and xylene prior to being embedded in paraffin. Subsequently, 5 μm sections were obtained from the paraffin-embedded specimens, stained with hematoxylin–eosin (H&E), and examined under a digital light microscope to evaluate the length of the intestinal villi.

### 2.6. Collection of fecal and intestinal contents and 16S rRNA sequencing

The microbial DNA from fecal and intestinal samples was extracted using the E.Z.N.A. Stool DNA Kit, with the purity of the extracted DNA verified through agarose gel electrophoresis. A paired-end library was then generated in accordance with Illumina’s genomic DNA library preparation protocols, utilizing the NEXTFLEX Rapid DNASeq Kit. The hypervariable V3–V4 regions of the 16S rRNA gene were amplified using the 338F and 806R primer sets. Subsequently, the amplification pyrosequencing was conducted on the Illumina HiSeq 2000 at Shanghai Majorbio Bio-pharm Technology Co., Ltd. in accordance with the manufacturer’s instructions. Raw sequencing data were subjected to quality control by Fastp, and the raw data were merged using FLASH. The operational taxonomic units were clustered on the basis of a 97% sequence identity threshold, utilizing UPARS. Taxonomic labels were assigned by the RDP classifier. Taxonomic classification was performed from the phylum to genus and species levels, with the UniFrac method being used for clustering the high-quality sequences in the R software package. Alpha diversity was calculated from the OTUs’ sparsity and Simpson’s index of evenness, while the beta diversity was compared through the UniFrac metric via principal coordinate analysis (PCoA).

### 2.7. Statistical analysis

A statistical analysis was performed using SPSS 20.0 software, and the results were presented as the mean ± SD (x ± s). The level of statistical significance was established at *p* < 0.05. To evaluate the statistical and biological differences, Graphpad 9 software was utilized, which used one-way ANOVA for parametric data and Kruskal–Wallis tests for non-normal data. The gut microbiota (GM) was analyzed through the application of the linear discriminant analysis effect size (LEfSe), while changes in the microbial communities before and after the treatments were visualized by partial least squares discriminant analysis (PLS-DA). The alpha diversity values were set at a threshold of 0.05, with a logarithmic score of linear discriminant analysis of ≥2.0 and a confidence level of 95% (*p* < 0.05).

## 3. Results

### 3.1. Intestinal section and diarrhea index

Figure 1B displays the results of the study, which indicated a statistically significant increase (*P* < 0.001) in the diarrhea activity index (DAI) for the model group. However, the administration of LCEO at low, medium, and high doses produced a significant reduction (*P* < 0.001) in the DAI compared with the model group. These findings suggested that LCEO might have potential as a therapeutic intervention for the management of diarrhea. Nonetheless, further investigations are warranted to delineate the underlying mechanisms of action by which LCEO mitigates DAI and to evaluate its safety and efficacy in clinical settings. To demonstrate the protective effects of LCEO on the intestine, an assessment of intestinal structural damage was also performed. In the LPS-treated model group, granulation tissue formed in the duodenum, accompanied by a large amount of villous desquamation in the ileum, a shortened villus length, and a reduced number of goblet cells, resulting in the formation of granulation tissue. The epithelial cells of the jejunum also desquamated, with villous rupture and leakage of the blood cells. Conversely, a significant improvement in LPS-induced pathological changes in the colonic tissue and partial restoration of the structure of glandular and goblet cells was observed at doses of 200 and 400 mg/kg LCEO, suggesting a role in protecting crypts and the epithelial integrity of the colon. The results demonstrated that LPS could cause damage to intestinal tissue’s morphology, while LCEO had a protective effect on intestinal tissue, with the highest efficacy observed at high doses of LCEO (Figure 1C). To further assess the impact of LCEO on inflammation, we investigated the cytokine levels in the liver and intestines. The low and medium doses of LCEO significantly lowered the levels of TNF-α, IL-6, and IL-1β in liver in LPS-treated mice (Figure 1D). The medium dose of LCEO also significantly decreased TNF-α, IL-1β, and IL-6 mRNA levels in the duodenum compared with those in the LPS group. Additionally, the low dose of LCEO also significantly decreased the mRNA levels of TNF-α and IL-1β in the duodenum compared with the LPS group. All the results showed the anti-inflammatory effect of LCEO, and the medium dose had the best effect (Figure 1E).

### 3.2. Molecular docking of LCEO

**Figure 2:**
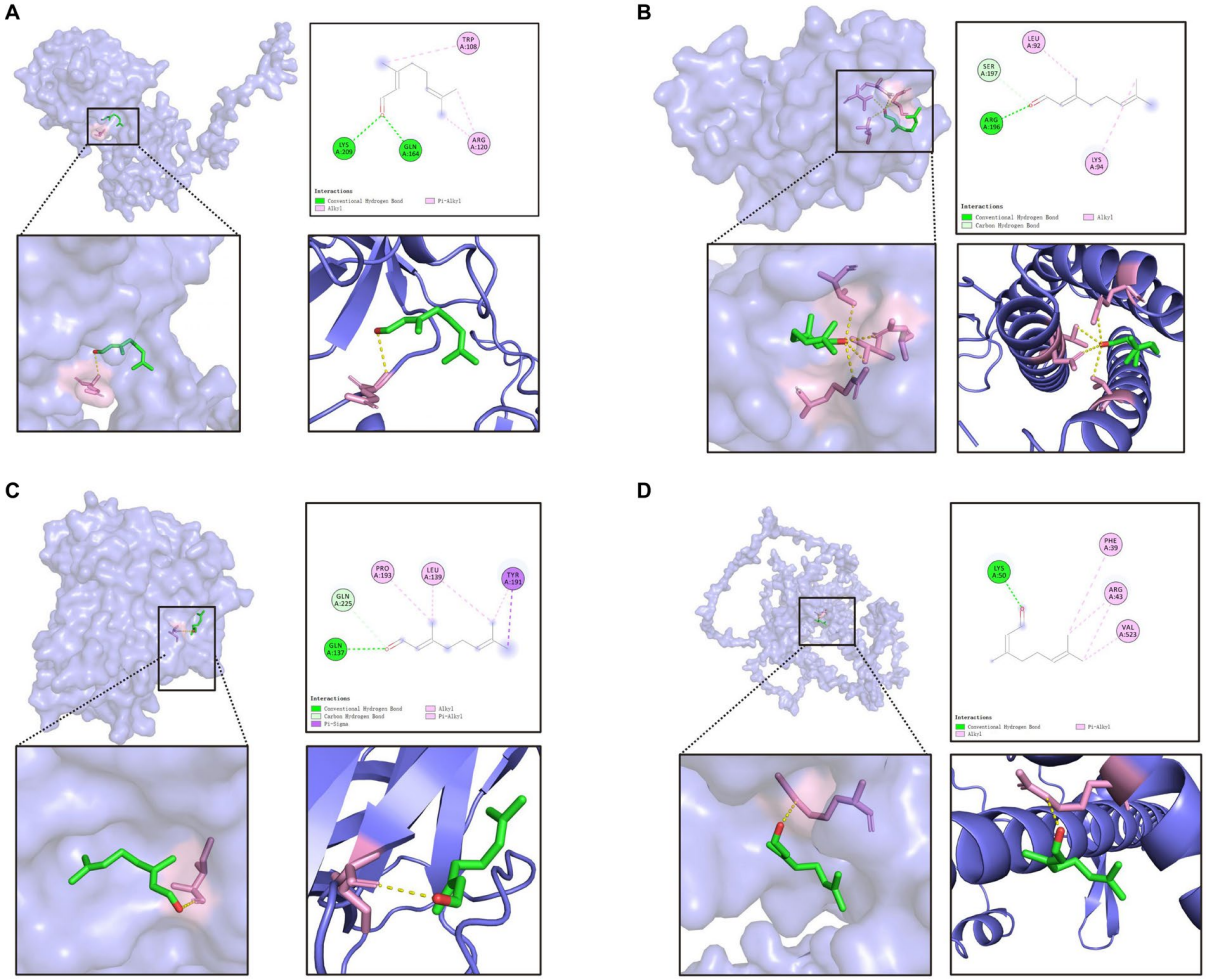
Molecular docking of LCEO **(A)** Molecular docking of citral with IL-1β. **(B)** Molecular docking of citral with IL-6. **(C)** Molecular docking of citral with TNF-α. **(D)** Molecular docking of citral with Nrf-2.

Table 2 lists the binding energies of nine different docking methods between citral and 4 inflammatory compounds. Binding energy indicates the affinity of the ligand to the receptor, and less than 0 kcal/mol indicates that these ligands and receptors can bind spontaneously. The lower the binding capacity, the more stable the binding of the ligand to the receptor. The docking result is shown in Figure 2. In molecular docking, the higher the absolute value of the binding energy, the more stable the binding. The molecular docking of citral and the inflammatory factors showed that citral can bind well with IL-1β, IL-6, TNF-α, and Nrf2, and citral has an anti-inflammatory function. Citral binds to the LYS-209 and GLN-164 of IL-1β through hydrogen bonds, binds to the ARG-196 of IL-6 through hydrogen bonds, binds to the SER-197 of IL-6 through a hydrophobic interaction, binds to the GLN-137 of TNF-α through hydrogen bonds, binds to the GLN-225 of TNF-α through a hydrophobic interaction, and binds to the LYS-50 of Nrf2 through hydrogen bonds (Figure 2).

**Table 2:** Molecular docking binding energy.

Ingredient	Chemical formula	Protein	Binding energy (kcal/mol)
Citral	C10H16O	IL-1β	−4.489
Citral	C10H17O	IL-1β	−4.283
Citral	C10H18O	IL-1β	−4.237
Citral	C10H19O	IL-1β	−4.175
Citral	C10H20O	IL-1β	−4.171
Citral	C10H21O	IL-1β	−4.141
Citral	C10H22O	IL-1β	−4.14
Citral	C10H23O	IL-1β	−4.125
Citral	C10H24O	IL-1β	−4.115
Citral	C10H25O	IL-6	−4.637
Citral	C10H26O	IL-6	−4.601
Citral	C10H27O	IL-6	−4.415
Citral	C10H28O	IL-6	−4.266
Citral	C10H29O	IL-6	−4.184
Citral	C10H30O	IL-6	−4.18
Citral	C10H31O	IL-6	−4.144
Citral	C10H32O	IL-6	−4.143
Citral	C10H33O	IL-6	−4.131
Citral	C10H34O	TNF-α	−3.759
Citral	C10H35O	TNF-α	−3.466
Citral	C10H36O	TNF-α	−3.416
Citral	C10H37O	TNF-α	−3.402
Citral	C10H38O	TNF-α	−3.343
Citral	C10H39O	TNF-α	−3.207
Citral	C10H40O	TNF-α	−3.042
Citral	C10H41O	TNF-α	−2.991
Citral	C10H42O	TNF-α	−2.98
Citral	C10H43O	Nrf2	−4.992
Citral	C10H44O	Nrf2	−4.45
Citral	C10H45O	Nrf2	−4.41
Citral	C10H46O	Nrf2	−4.37
Citral	C10H47O	Nrf2	−4.348
Citral	C10H48O	Nrf2	−4.334
Citral	C10H49O	Nrf2	−4.311
Citral	C10H50O	Nrf2	−4.3
Citral	C10H51O	Nrf2	−4.283

### 3.3. Analysis of the intestinal microbial community after the essential oil intervention

**Figure 3:**
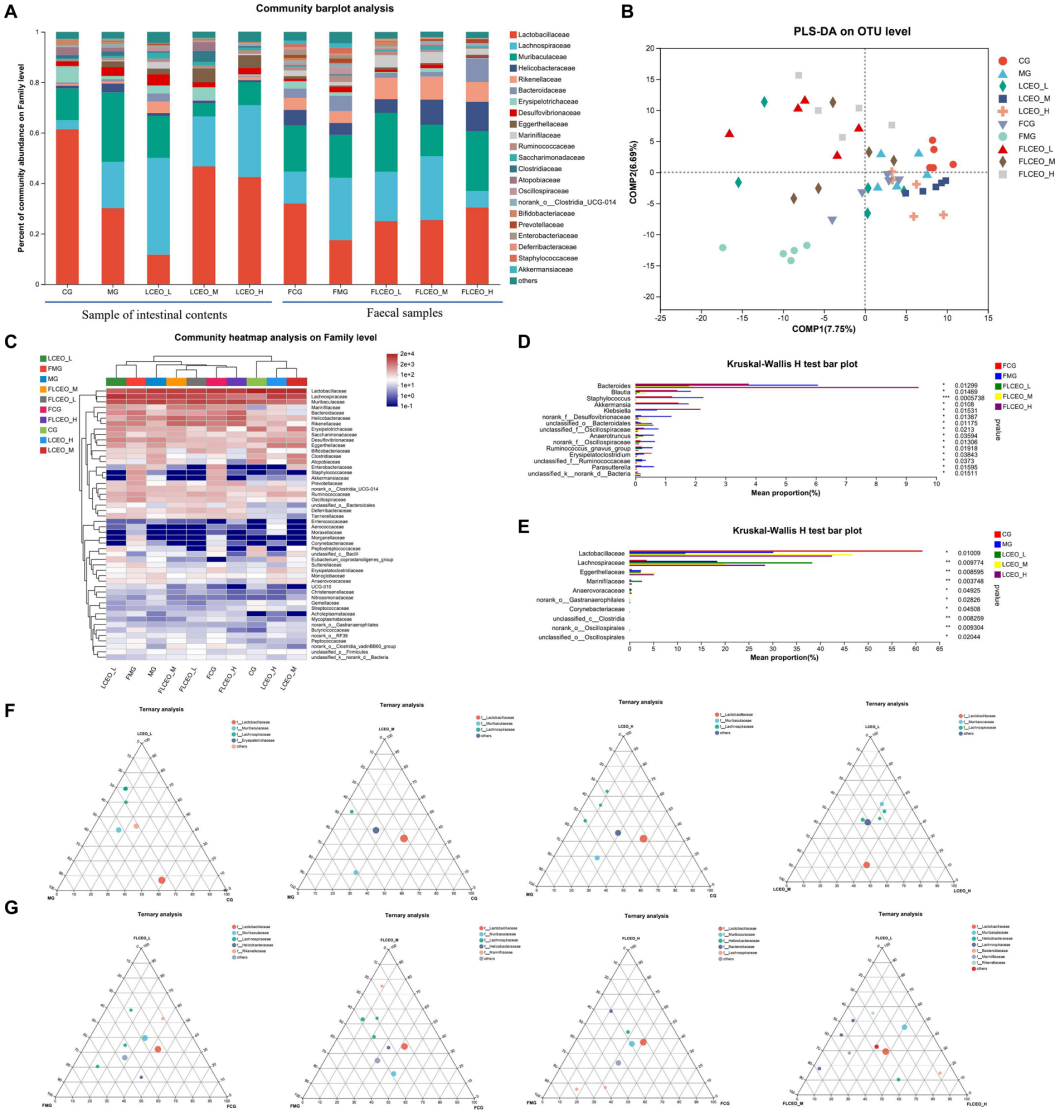
Composition of the gut microbiota and fecal samples. **(A)** bacterial taxonomic compositions at the levels of genus in the intestinal digesta and the feces. **(B)** PLS-DA score plots. **(C)** Community heatmap analysis on Family level. **(D)** KrusKal-Wallis H test bar plot of fecal samples. **(E)** KrusKal-Wallis H test bar plot of intestinal content. **(F)** Ternary analysis of intestinal content. **(G)** Ternary analysis of fecal samples.

The community barplot analysis elucidated pronounced disparities in microbiological constitution between fecal samples procured prior to LPS injection, and intestinal content samples succeeding LPS injection. Nonetheless, the deviation in microbiological composition between fecal samples and the intestinal content of the control group was marginal (Figures 3A–C). Within the quintet of fecal material groups, an array of microbial taxa, encompassing *Bacteroides*, *Blautia*, *Staphylococcus*, *Akkermansia*, *unclassified_k_norank_d_Bacteria*, *Parasutterella*, *unclassified_f_Ruminococcaceae*, *Erysipelatoclostridium*, *Ruminococcus_gnavus_*group, *norank_f__Oscillospiraceae*, *anaerotruncus*, *unclassified_f__Oscillospiraceae*, *unclassified_o_Bacteroidales*, and *norank_f__Desulfovibrionaceae*, demonstrated considerable disparities. It is noteworthy that *Staphylococcus*, *Akkermansia*, and *Klebsiella* were conspicuously absent in the LCEO cohorts. In contrast, the essential oil intervention group exhibited a substantial upsurge in *Lactobacillaceae*, *Lachnospiraceae*, *Eggerthellaceae*, and *Marinifilaceae* (Figures 3D,E).

In pursuit of delineating the effects of essential oils on intestinal flora, a ternary diagram was leveraged, elucidating variations in community structure abundance at the family echelon among distinct treatment groups (control, model, and essential oil). Each dot embodied an OTU, with its size, hue, and position denoting the relative abundance, taxonomy, and cluster classification of the OTUs, correspondingly. Scrutiny of gut contents manifested pronounced disparities in flora abundance between the trinity of essential oil doses and the model and control groups (Figures 3F,G). The MG group manifested the utmost abundance of *Bacteroidales*, whereas the CG group disclosed heightened prevalence of *Lactobacillales*, accompanied by *Lachnospiraceae* bacteria in the LCEO-L group, *Lactobacillaceae* in the CG group, and *Muribaculaceae* bacteria in the MG group. Notably, no explicit bacterial predilection was discernible among the trio of essential oil groups. An ensuing ternary analysis was undertaken using fecal samples, which presented a more intricate composition. However, marked differences in fecal flora were discernible between the essential oil cohorts and the model and control groups. In comparing disparate doses of essential oils, the FLCEO-L group was typified by heightened abundance of *Rikenellaceae*, the FLCEO-M group by abundant *Lachnospiraceae* and *Marinifilaceae*, while the FLCEO-H group by prevalence of *Helicobacteraceae* and *Bacteroidaceae*. In contrasting the essential oil and control groups with the model group, the control group exhibited diminished abundance of associated flora in their feces, whereas the model group revealed a heightened abundance of *Lachnospiraceae* and *Lachnospiraceae*. These findings underscore significant discrepancies in the intestinal microbial community structure between intestinal contents and fecal matter.

### 3.4. Analysis of the association between the gut microbiota and factors of intestinal inflammation

**Figure 4:**
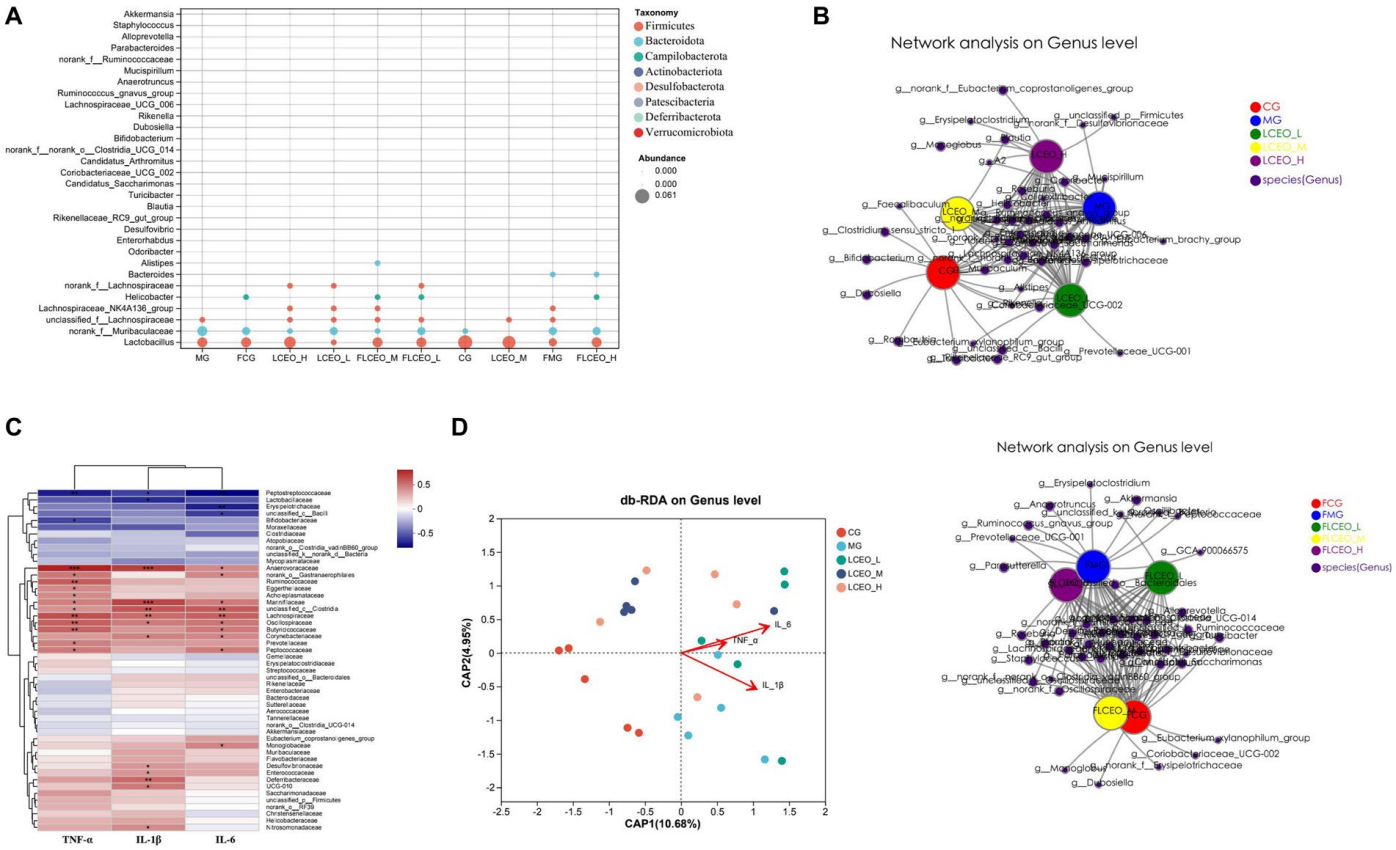
**(A)** The composition of the intestinal and fecal samples in bubble chart. **(B)** The network analysis on genus level intestinal and fecal samples. **(C)** Correlation heatmap between inflammatory factors and selected species on Genus and Species level. **(D)** RDA/CCA analysis of the relationship between microbiota and inflammatory factor.

Our preliminary analysis of the flora in the feces and intestinal contents using bubble plots revealed that three genera, namely *Lachnospiraceae*, *Muribaculaceae*, and H*elicobacter*, were more affected in both sample types. In the intestinal contents, the abundance of *Lachnospiraceae* was significantly higher in the essential oil groups than in the other groups, indicating that essential oils had an important effect on the abundance of *Lachnospiraceae*. In contrast, the abundance of *Muribaculaceae* was inhibited by the intervention of essential oils, particularly in the intestinal contents (Figure 4A). By creating co-occurrence network diagrams to visualize the co-occurrence of the species in the different samples, we confirmed the differences in the effects of different doses of essential oils on the gut microbiota (Figure 4B). The present study aimed to explore the intricate relationships between inflammatory factors and selected species at both the genus and species level. To achieve this, we used a correlation heatmap analysis, which revealed that the correlation between the two was dynamic, with some interactions exhibiting a positive correlation (depicted in blue) and others showing a negative correlation (depicted in red) (Figure 4C). This analysis was performed to gain more insight into the protective effects of essential oil on the gut microbiota and the parameters of LPS-induced inflammation in mice. The result showed that the potential correlations between the gut microbiota at the species level and the expression levels of specific genes, including IL-1β, IL-6, and TNF-α in the duodenum. The results showed that the groups of bacteria that showed a negative correlation with inflammatory factors were *Peptostreptococcaceae, Lactobacillaceae, Erysipelotrichaceae,* and *Bifidobacteriaceae*. Conversely, the groups that showed a positive correlation with inflammatory factors were *Anaerovoracaceae, Ruminococcaceae, Eggerthellaceae, Acholeplasmataceae, Marinifilaceae, Clostridia, Lachnospiraceae, Oscillospiraceae Butyricicoccaceae, Corynebacteriaceae, Prevotellaceae, Peptococcaceae, Monoglobaceae, Desulfovibrionaceae, Enterococcaceae, Deferribacteraceae,* and *UCG-010*. Overall, this analysis provided a comprehensive understanding of the intricate relationships between inflammatory factors and selected species on the genus and species level, thus emphasizing the importance of considering both factors in the context of inflammation. In addition, we used a redundancy analysis (RDA) or canonical correspondence analysis (CCA) to analyze the complex interplay between the microbiota and environmental factors. The RDA/CCA analysis had red arrows to represent the quantitative environmental factors, with the length of each arrow reflecting the degree of the environmental factor’s influence on the species. The angles between the arrows of the environmental factors were used to signify positive and negative correlations, with an acute angle indicating a positive correlation, an obtuse angle signifying a negative correlation, and a right angle implying no correlation between the environmental factors and the species. The results showed that IL-1β, IL-6, and TNF-α were positively related to each other, and that the MG group was greatly affected by inflammatory factors (Figure 4D).

### 3.5. Specific bacterial taxa in the feces and intestinal contents

**Figure 5:**
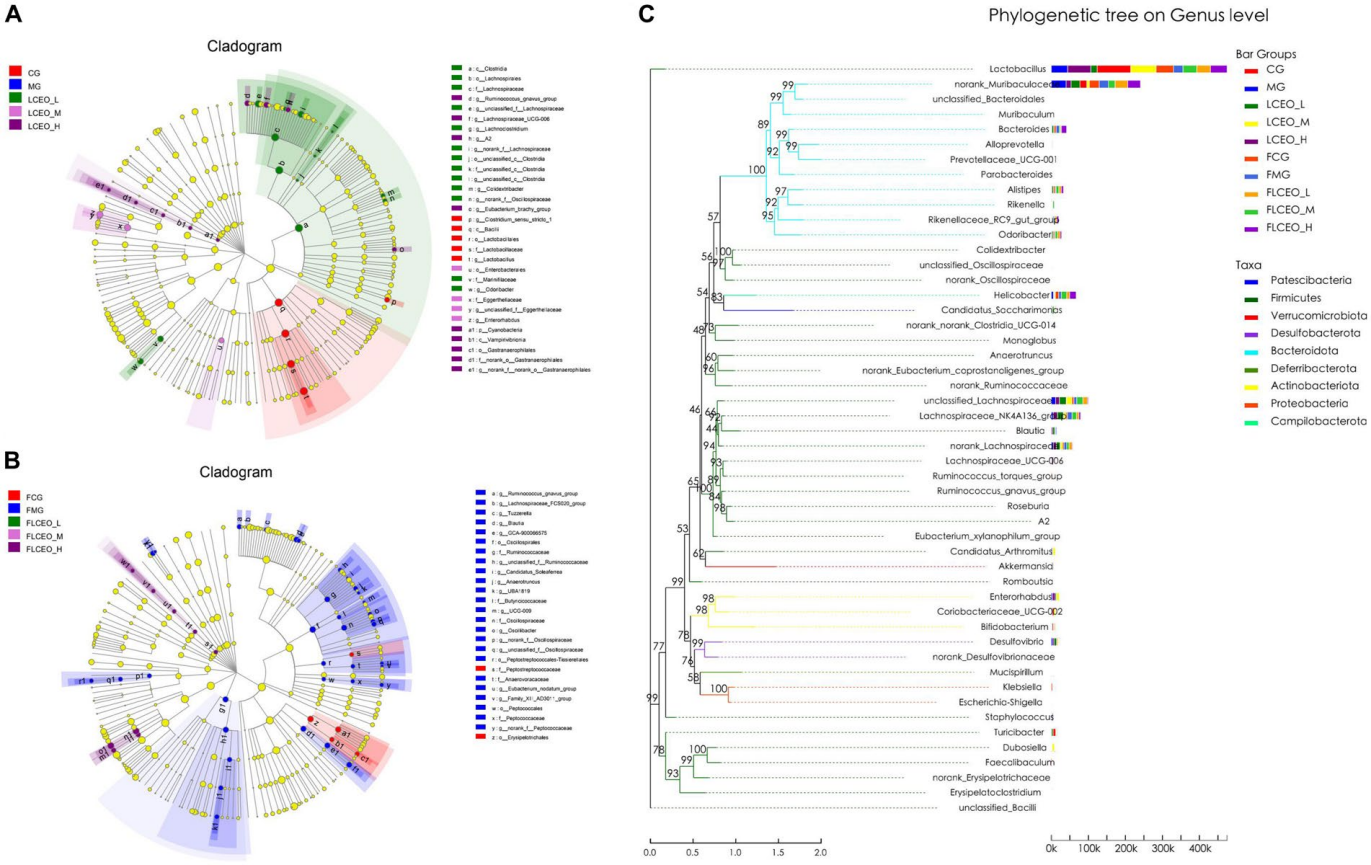
The specific microbiota taxa in samples **(A)** Species with significant differential effects at different taxonomic levels in LEfsa analysis in intestinal. **(B)** Species with significant differential effects at different taxonomic levels in LEfsa analysis in fecal. **(C)** polygenetic tree on genus level.

The histogram depicting the distribution of the LDA values and the evolutionary branch diagram were meticulously crafted through the LDA effect size (LEfSe) analysis. These insightful visualizations enabled the exploration of biomarkers, showcasing statistical disparities among the experimental cohorts. The taxonomic cladogram of LEfSe offers a captivating portrayal of the pivotal bacterial modifications, using diverse hues to represent the distinct groups, while the sizes of the circles reflect their relative abundance. The outcomes revealed notable divergence in the composition of the gut microbiota among the different groups. Notably, the LEfSe analysis successfully discerned numerous genera that serve as noteworthy biomarkers, delineating taxonomic discrepancies among the five cohorts. In the intestinal contents samples, 16 genera were identified as discriminating biomarkers, one in the CG group (*Lactobacillus*), eight in the LCEO-L group (*Ruminococcus_gnavus_group, unclassified_f_Lachnospiraceae, Lachnoclostridium, norank_f_Lachnospiraceae unclassified_c_Clostridia, Colidextribacter, norank_f_Oscillospiraceae,* and *Odoribacter*), two in the LCEO-M group (*unclassified_f_Eggerthellaceae,* and *Enterorhabdus*), five in yjr LCEO-H group (*Ruminococcus_gnavus_group, Lachnospiraceae_UCG-006, A2, Eubacterium_brachy*_*group*, and *norank_f_norank_o_Gastranaerophilales*) (Figure 5A). Additionally, 16 genera were identified as discriminating biomarkers only in the fecal samples (*Ruminococcus_gnavus_group, Lachnospiraceae_FCS020_group, Tuzzerella, Blautia, GCA-900066575, unclassified_f_Ruminococcaceae, Candidatus_soleaferrea, Anaerotruncus, UBA1819, UCG-009, Oscillibacter, norank_f_Oscillospiraceae, unclassified_f_Oscillospiraceae, Eubacterium_nodatum_group, Family_XIII_AD3011_group,* and *norank_f_Peptococcaceae*) (Figure 5B). Subsequently, we performed an evolutionary tree analysis of the gut microbes and found that *Lactobacillus* was the most abundant of all groups, followed by *Muribaculaceae* (Figure 5C).

## 4. Discussion

The therapeutic potential of essential oils, especially their role in inflammation modulation, is well-documented. In a rigorous analysis, mice subjected to geranium oil treatment showed an increased incidence of yeast-form cells in vaginal smears, indicating the potential of geranium oil and its component, geraniol, to prevent vaginal candidiasis. *In vitro* evaluations revealed geranium oil and geraniol’s efficacy in inhibiting fungal filament growth at a modest concentration (25 μg/mL), despite a lack of impact on yeast growth (Maruyama et al., 2008). Concurrently, kanuka and manuka essential oils demonstrated potent antimicrobial and anti-inflammatory properties, suggesting potential utility as pharmaceutical antibiotics, cosmeceutical agents, and dietary supplements (Chen et al., 2016). Furthermore, investigations into the biological activities of 10 essential oils (nine individual and one blend) within a human dermal fibroblast system simulating chronic inflammation revealed notable anti-inflammatory, tissue remodeling, and immunomodulation effects. These findings reinforce the therapeutic potential of essential oils as potential adjunct treatments for a range of conditions (Han et al., 2017).

Historically, the essential oil derived from *L. cubeba* has primarily been investigated for its anti-inflammatory and antibacterial attributes. Notably, Lin elucidated the oil’s potential prophylactic capabilities in mitigating rheumatoid arthritis, employing a rat model with Freund’s complete adjuvant-induced arthritis (Lin et al., 2013). The consequential discoveries underscored a substantial mitigation of arthritis via downregulation of pro-inflammatory cytokines and inflammatory enzymes, concurrently with an increase in anti-inflammatory cytokine IL-10 levels, thereby implying prospective applications of *L. cubeba* roots in human arthritis therapeutics. Our research subsequently emphasizes LCEO’s impact on gastrointestinal health. Our analysis revealed the oil’s potential as a treatment for diarrhea and its protective effect on gut integrity, with evidence of reduced intestinal tissue damage and maintained crypt and colon epithelial integrity. Moreover, the oil displayed anti-inflammatory characteristics, as evidenced by reduced TNF-α, IL-6, and IL-1β levels in hepatic and intestinal tissues. A prior study on the antibacterial action and kinetics of LCEO identified aldehydes—comprising about 70% of the total constituents—as the key contributors to its antibacterial efficacy, with minor components synergistically enhancing this effect. Therefore, given its antimicrobial properties, *L. cubeba* oil exhibits broad utility in industries ranging from flavoring and spicing to antimicrobial interventions (Li et al., 2014).

Previous research has shed light on the significant interactions between citral, a major component of *Litsea cubeba* essential oil, and the Nrf2 pathway. For instance, a study by Ka et al. showed that citral could ameliorate the severity of lupus nephritis by enhancing Nrf2 activation and inhibiting the activation signal of the NLRP3 inflammasome (Ka et al., 2015). Another research conducted by Yang et al. highlighted the renoprotective effect of citral in a model of focal segmental glomerulosclerosis, partly attributed to the activation of the Nrf2 pathway (Yang et al., 2013). Furthermore, a recent study demonstrated the protective effects of citral against LPS-induced endometritis, finding that these effects were exerted through inhibiting ferroptosis and activating the Nrf2 signaling pathway (Zhao et al., 2023). Given these compelling evidences of citral’s interaction with the Nrf2 pathway, and the pivotal role this pathway plays in regulating oxidative stress and inflammation, we selected it as a primary focus of our research. This added context underpins our investigation into the impacts of LCEO, and particularly citral, on gut health. Citral, as the main natural compound of LCEO, which is also found in citrus fruits and essential oils such as lemongrass and lemon verbena, possesses various beneficial properties. One notable function of citral is its potential anti-inflammatory activity. We also found that citral exhibited anti-inflammatory effects by modulating inflammatory factors involved in the immune response. In our study, citral showed strong binding to IL-1β, IL-6, and TNF-α, and exerted anti-inflammatory effects via hydrogen-bonding interactions. It was found to bind effectively to inflammatory factors such as interleukin-1β (IL-1β), interleukin-6 (IL-6), and tumor necrosis factor-alpha (TNF-α), which were key mediators in the inflammatory process. Through these hydrogen-bonding interactions, citral interacts with specific amino acid residues in these inflammatory factors, thereby inhibiting their activity. By binding to IL-1β, IL-6, and TNF-α, citral may interfere with the signaling pathways that promote inflammation, thereby reducing the production and release of pro-inflammatory cytokines. Overall, citral shows promise as a natural compound with anti-inflammatory properties. Moreover, some researchers have investigated its *in vitro* effects on the production of cytokines (IL-1β, IL-6, and IL-10) both before and after LPS incubation, demonstrating the anti-inflammatory effects and potential role of citral in inhibiting the production of cytokines through suppression of the transcription factor NF-KB (Bachiega and Sforcin, 2011). In addition, citral was found to activate PPAR-γ, and its anti-inflammatory effects were reversed by the PPAR-γ antagonist GW9662. Using a well-established rat model of systemic LPS administration, Ka et al. (2015) reported the *in vivo* antipyretic effects of citral, suggesting that its protective mechanism in the mouse ASLN model involved inhibiting the activation of the NLRP3 inflammasome signal and enhancing the Nrf2 antioxidant signal. Muñoz-Pérez (Munoz-Perez et al., 2021) further investigated the modulatory role of citral in inflammation resulting from infection with *S. aureus* and discovered a significant inhibition of PGF-2α-induced contractions, a concentration-dependent increase in myometrial cAMP levels, and a concentration-dependent decrease in the LPS-induced production of TNFα and IL-1β, while the production of IL-10 was significantly increased. In conclusion, according to the findings of others and the present study, we have reason to believe that citral plays a key role in alleviating LPS-induced intestinal inflammation in mice, and that these studies have contributed to a comprehensive understanding of the potential therapeutic applications of citral and the underlying mechanisms of various biological processes.

In addition to its inflammatory effect, essential oils have an important impact on the abundance of intestinal flora and bacteria. Previous studies found that a mixture of organic acids and essential oils had a beneficial effect on the gut microflora and improved the immune response and disease resistance of *L. vannamei* (He et al., 2017). In another study, the researchers evaluated the effects of three essential oils, namely cinnamon bark oil (CNO), clove bud oil (CLO), and ajwain seed oil (AJO), with different major chemical structures as alternatives to antibiotic growth promoters (AGPs) on the gut health, immune response, and antioxidant status of broiler chickens (Chowdhury et al., 2018). In our research, we used community barplot analysis to discern significant disparities in the microbiological composition within fecal samples and intestinal contents before and after the essential oil intervention. Prominent variations in the taxa were identified, revealing marked differences in the abundance of flora among the essential oil groups, the model group, and the control group. The MG group displayed the highest abundance *of Bacteroidales*, the CG group exhibited the most substantial abundance of *Lactobacillales*, and the LCEO-L group was characterized by the dominance of *Lachnospiraceae*. Analyses of the fecal flora revealed heterogeneity among the essential oil groups, the model group, and the control group. When we compared the different doses of essential oil, FLCEO-L had increased *Rikenellaceae,* FLCEO-M had an abundance of *Lachnospiraceae* and *Marinifilaceae*, and FLCEO-H had an abundance of *Helicobacteraceae* and *Bacteroidaceae*. The control group had a reduced abundance of flora in the feces, whereas the model group had an increased abundance of *Lachnospiraceae*. Our findings unveil significant alterations in the composition of gut microbiota consequent to the administration of essential oils, underscoring the substantial variations observed between intestinal contents and feces. Essential oils were shown to augment the population of *Lachnospiraceae* in the intestinal contents while inhibiting *Muribaculaceae*. This demonstrates the substantial influence these oils may exert on intestinal microbial communities. Further, these modulations were observed to be dose-dependent, corroborated by co-occurrence networks. The discovery of bacterial groups inversely correlated with inflammation, such as *Peptostreptococcaceae, Lactobacillaceae, Erysipelotrichaceae*, and *Bifidobacteriaceae,* as well as those showing positive correlation, like *Anaerovoracaceae, Ruminococcaceae*, and *Eggerthellaceae,* suggests an intriguing potential for essential oils to modulate inflammation via microbiota manipulation. Future exploration could delve into investigating this anti-inflammatory potential and the precise mechanisms of action. LEfSe analysis revealed discriminatory biomarkers, including the genera *Lactobacillus, Ruminococcus_gnavus_*group, and *Lachnoclostridium* among others. Notably, *Lactobacillus*, the most abundant genus, and *Muribaculaceae* revealed distinct divergence in their distributions within the evolutionary tree, presenting potential targets for future investigation. The stark divergence in the gut microbiota composition demands further exploration to elucidate the possible impacts on host health and disease resistance. The findings of this study suggest that essential oils can cause significant changes in the composition of gut microbiota. Importantly, essential oils were found to increase the population of Lachnospiraceae while decreasing Muribaculaceae. This suggests that essential oils may have a significant influence on the balance of microbial communities within the intestine, and these changes appear to be dose-dependent. Another key aspect of this research was the discovery of bacterial groups that correlated negatively with inflammation, including *Peptostreptococcaceae, Lactobacillaceae*, *Erysipelotrichaceae*, and *Bifidobacteriaceae*, as well as bacterial groups that correlated positively with inflammation, such as *Anaerovoracaceae*, *Ruminococcaceae*, and *Eggerthellaceae*. This finding could have significant implications for the development of essential oil-based treatments or preventive strategies for inflammatory diseases, suggesting a potential mechanism through which essential oils might exert their effects by modulating the gut microbiota.

Overall, this study propounds a comprehensive understanding of the potential applications and mechanisms of essential oils in various animal production settings. However, the far-reaching implications of these findings go beyond the realm of animal health. Given the burgeoning interest in the human gut microbiome’s role in health and disease, our insights could pioneer new avenues for employing essential oils in manipulating gut microbiota for promoting human health, with possible applications in disease treatment, health maintenance, and even personalized nutrition. In this study, we explored the effects of LCEO on LPS-induced intestinal inflammation and the gut microbiome. Our findings demonstrated that the LCEO treatment alleviated gut inflammation and suggested its potential in the treatment of intestinal inflammation. However, there are certain limitations to consider in our study. Firstly, our study focused on the effects of LCEO on LPS-induced intestinal inflammation in experimental models. Further investigations are needed to evaluate its efficacy in clinical settings, as well as its safety profile. Secondly, while we observed changes in the composition of the gut microbiome following the LCEO intervention, our study only provided a snapshot of the microbial community at a specific time point. Longitudinal studies would provide a more comprehensive understanding of the dynamic changes in the gut microbiome over time. Thirdly, the mechanisms underlying the anti-inflammatory effects of LCEO and its interaction with the gut microbiome remain to be fully elucidated. Additional research is warranted to explore the specific pathways and molecular mechanisms involved in the observed effects. Moreover, our study focused on the evaluation of citral as the main natural compound in LCEO. While citral has demonstrated anti-inflammatory properties, further studies are needed to investigate its specific mechanisms of action and its potential as a therapeutic agent. Lastly, our study primarily relied on animal models and *in vitro* experiments. The translation of these findings to human subjects may vary, and clinical studies are needed to validate the efficacy and safety of LCEO in human populations. In conclusion, while our study provides valuable insights into the anti-inflammatory effects of LCEO and its impact on the gut microbiome, further research is necessary to address the limitations and fully understand the therapeutic potential of LCEO in the management of intestinal inflammation.

## Data availability statement

The datasets presented in this study can be found in online repositories. The names of the repository/repositories and accession number(s) can be found in the article/supplementary material.

## Ethics statement

In accordance with the established guidelines of the Animal Ethics Committee at Hunan Heyuan Biotechnology Co., Ltd. (LCH2022015).

## Author contributions

LX: writing—original draft, methodology, data curation, visualization, software, formal analysis, resources, investigation, and validation. TT: methodology, validation, and writing—review and editing. HD: methodology and resources. RZ and ZL: methodology, validation, and writing—review and editing. RL, ZS, and CX: conceptualization, methodology, writing—review and editing, supervision, project administration, and funding acquisition. All authors contributed to the article and approved the submitted version.

## Funding

This work was supported by the Research and Development Plan in Key Fields of Hunan Province (2022SK2126), Research Foundation of Hunan Administration of Traditional Chinese Medicine (A2023054, 2019101), Shaoguan University Doctoral Research Fund (142-9900064602), Research Foundation of Natural Science Foundation of Hunan Province (2022JJ30041, 2021JJ40550, 2022JJ80098, and 2022JJ50101), Scientific Research Project of Hunan Provincial Health Commission (202103101455 and 202102081835), Clinical Innovation Leading Scientific Research Projects of Hunan Science and Technology Department (2020SK52603 and 2021SK52901), Guangdong Basic and Applied Basic Research Foundation (2022A1515111127), Science and Technology Program of Guangzhou (2023A04J0468), and China Postdoctoral Science Foundation (2022M710908).

## Conflict of interest

The authors declare that the research was conducted in the absence of any commercial or financial relationships that could be construed as a potential conflict of interest.

## Publisher’s note

All claims expressed in this article are solely those of the authors and do not necessarily represent those of their affiliated organizations, or those of the publisher, the editors and the reviewers. Any product that may be evaluated in this article, or claim that may be made by its manufacturer, is not guaranteed or endorsed by the publisher.
