# Corrigendum: Therapeutic potential of *Litsea cubeba* essential oil in modulating inflammation and the gut microbiome

**DOI:** 10.3389/fmicb.2025.1610268

**Published:** 2025-04-25

**Authors:** Liqiong Xia, Ran Li, Ting Tao, Ruimin Zhong, Haifang Du, Ziling Liao, Zhanghua Sun, Changqiong Xu

**Affiliations:** ^1^Department of Pharmacy, Loudi Central Hospital, Loudi, Hunan, China; ^2^Guangdong Provincial Key Laboratory of Utilization and Conservation of Food and Medicinal Resources in Northern Region, Shaoguan University, Shaoguan, Guangdong, China; ^3^Hunan Yueyang Maternal and Child Health-Care Hospital, Yueyang, Hunan, China; ^4^The Second Clinical Medical College, Guangzhou University of Chinese Medicine, Guangzhou, China; ^5^Medical College of Shaoguan University, Shaoguan, Guangdong, China

**Keywords:** inflammation, essential oils, gut microbiome, LPS, *Litsea cubeba*, citral

In the published article, there was an error. The wrong animal ethics committee was stated.

A correction has been made to **2. Methods**, *2.2 Experimental design and induction of colitis*, Paragraph 1.

This sentence previously stated:

“In accordance with the established guidelines of the Animal Ethics Committee at Loudi Central Hospital (LCH2022015),”

The corrected sentence appears below:

“In accordance with the established guidelines of the Animal Ethics Committee at Hunan Heyuan Biotechnology Co., Ltd. (LCH2022015),”

The authors apologize for this error and state that this does not change the scientific conclusions of the article in any way. The original article has been updated.

